# Key factors influencing long-term engagement: a quantitative analysis of Special Olympics World Games 2023 volunteers

**DOI:** 10.3389/fspor.2025.1503863

**Published:** 2025-02-26

**Authors:** Antonia Hannawacker, Lina-Doreen Rose, Holger Preuss

**Affiliations:** Institute of Sports Science, Johannes Gutenberg University, Mainz, Germany

**Keywords:** sport event volunteering, volunteering Special Olympics, social legacy, volunteering legacy, long-term engagement, Special Olympics World Games, VMS-ISE

## Abstract

**Introduction:**

The contribution of volunteers to the success of mega sport events is very valuable, as they dedicate their time and skills without financial compensation. Despite the undiminished enthusiasm for volunteering at events, grassroots sport in Europe faces increasing challenges in retaining long-term volunteers in sports clubs. This study aims to contribute to existing research by examining the characteristics and motivations of volunteers at mega sport events, with a particular focus on the Special Olympics World Games 2023 (SOWG 23). Furthermore, it seeks to identify factors that influence long-term engagement, ultimately promoting a sustainable volunteering legacy.

**Methods:**

A quantitative methodology was employed, utilizing a structured questionnaire survey conducted in the context of the SOWG 23 in Berlin. The questionnaire addressed various domains, including satisfaction, expectations, volunteering history, future volunteering intentions, perceptions of sport event characteristics, and socio-demographic variables. Additionally, an extended version of the VMS-ISE scale was employed in order to ascertain the motives of the surveyed volunteers. Following verification, 512 validated responses were subjected to analysis employing descriptive and inferential statistical methods and techniques.

**Results:**

The results demonstrate that most respondents were female, married or in life partnerships, without children, of advanced age, highly educated, and with prior volunteering experience despite no parental history of volunteering. Intrinsic motivations were pivotal for volunteer engagement at the SOWG 23, while extrinsic rewards had a lesser influence. The group-specific analysis, based on individual volunteering history, identified key factors that distinguished those who became active as a result of the event from those who did not. The binomial logistic regression model developed was statistically significant [*χ*^2^(18) = 48.01, *p* < .001] and explained a large proportion of the variance (*R*^2^ = .51).

**Discussion/Conclusion:**

This study enhances the understanding of the characteristics and motivations of volunteers engaged in the Special Olympics context, and identifies specific factors that facilitate long-term engagement. Furthermore, the findings offer invaluable insights that have informed the development of recruitment strategies aimed at fostering a volunteer legacy in both the sports and non-sports sectors.

## 1 Introduction

The success of major sport events heavily relies on the contribution of volunteers, who provide their skills, knowledge, time, and effort to ensure the event's success (1). These individuals are therefore a highly valuable human resource (2). The International Labour Office (3) defines volunteering as an “unpaid, non-compulsory work where individuals contribute time without remuneration to activities either organized by an entity or directly for the benefit of others outside their own household.” This emphasizes the voluntary nature, lack of financial compensation, and broader social benefits of volunteering.

The crucial role of volunteers at mega sport events (MSEs) is exemplified by those held this year. 45,000 volunteers were engaged for the 2024 Olympic and Paralympic Games in Paris (4) and approximately 16,000 volunteers were involved in the 2024 UEFA European Championship (5). These figures, in conjunction with the considerable number of applications, 146,000 for UEFA EURO 2024 alone (5), illustrate the considerable public enthusiasm for volunteering for MSEs.

This enthusiasm is also evident in the context of sport events for athletes with disabilities, where the contribution of volunteers is of particular importance. In addition to fulfilling organizational responsibilities, they play a crucial role in creating a secure environment that allows athletes to focus on their performance without concerns about their safety (6). To illustrate, 20,000 volunteers registered to donate their services for the Special Olympics World Games 2019 in Abu Dhabi (7). Furthermore, the Special Olympics World Games 2023 (SOWG 23) in Berlin necessitated the involvement of approximately 18,000 volunteers, with 22,000 individuals applying (8).

Despite the evident enthusiasm for volunteering at MSEs, long-term volunteerism in sports clubs and organizations is in decline. A notable decrease in the number of volunteers has been observed in recent years (9, 10). This is a cause for concern, particularly given that grassroots sports in Europe rely on volunteer support. In Germany, for instance, the “German Volunteer Survey 2014–2019” recorded a nearly 10% decline in volunteers at sports clubs over a five-year period (9).

Although there is extensive research on volunteering, including motivations, challenges and long-term impacts (11–15), it is not possible to apply these findings directly to the sport sector, due to the fact that episodic volunteers (i.e., at events) differ from traditional volunteers (16). Most studies of sport volunteering focus on individual motivations and experiences (17), particularly in relation to *episodic volunteers* who engage in event-based activities on a temporary basis (18–22).

This study aims to advance existing research on the characteristics and motivations of volunteers at MSEs, with a particular focus on SOWG 23, to identify the factors that influence their continued commitment to volunteering. The SOWG 23 provides a unique context, as it involves a large number of volunteers who are already actively engaged and have a deeper, more personal connection to the cause, as many are friends or family members of athletes (23). The objective of this study is, therefore, to address the following research question: Who are the volunteers of the Special Olympics World Games 2023, what motivates them, and which factors influence their long-term engagement in volunteering?

## 2 Literature review

### 2.1 Sport event volunteering in general

The extant literature on volunteering at sport events is primarily concerned with the characteristics, motivation and levels of satisfaction of volunteers at MSEs. This is illustrated by the following studies, with a particular focus on the Olympic Games.

In one of the earliest studies which examined the experiences of volunteers, Elstad (24) found that the majority of respondents expressed high levels of satisfaction in the context of the Olympic Winter Games in Lillehammer 1994. Key satisfaction factors included personal networking, the celebratory atmosphere, job-related competence, welfare issues, and job characteristics. Building on the existing literature on volunteer motivation, Dickson et al. (18) used an online questionnaire based on the Special Event Volunteer Motivation Scale (SEVMS) to explore volunteer motivations at the Vancouver Olympic and Paralympic Games in 2010. The results showed that the centrality of the Games was the primary motivating factor for the volunteers surveyed. In contrast, factors such as skill development and job enhancement were of minimal importance for the respondents, who were predominantly female, aged between 45 and 64 years, and employed in full-time positions. In 2014, Dickson et al. expanded their research by comparing volunteer motivations at the Vancouver 2010 and London 2012 Olympic and Paralympic Games, utilizing an adapted version of the SEVMS. The results showed that the demographic characteristics of surveyed volunteers were similar at both events, with the majority being female, over 35 years old, employed full-time or part-time, and having prior volunteering experience. The underlying motivations for participation in the events were also found to be consistent, including a desire to take part in a unique event, a commitment to the success of the Games, and a wish to contribute to the community. Although the SEVMS has been a commonly employed instrument for investigating motivations among volunteers at MSEs, the Volunteer Motivation Scale for International Sporting Events (VMS-ISE) has become the most comprehensive and widely used instrument for assessing the motivation of volunteers in sport in recent years, according to Angosto et al. (25). The study, which was conducted using a systematic literature review approach, encompassed an analysis of 30 studies that had been published between 2007 and 2021. The analysis concentrated on the motivations and future intentions of volunteers in the context of sport events. In addition to the findings regarding the VMS-ISE, it is noteworthy that among the motivational factors, the expression of values was identified as the most significant motive, followed by love of sport. Conversely, extrinsic rewards and career motivations were classified as less pertinent. A study illustrating the application of the VMS-ISE was conducted by Vetitnev et al. (26). An adapted version of the scale was used to analyze the motivation of volunteers at the 2014 Winter Olympics in Sochi. The results demonstrate a positive correlation between volunteer satisfaction and the motivational factors of expression of values, career orientation, and extrinsic rewards. Additionally, a positive correlation was observed between volunteer satisfaction and attitude toward the Games. Conversely, both commitment and tradition demonstrated a negative impact on volunteer satisfaction. In a recent contribution to the field, Teixeira et al. (21) employed semi-structured questionnaires to examine volunteers at the 2016 Rio de Janeiro Olympic Games, collecting a total of 828 responses. The results of the survey showed a prevalence of volunteers from Brazil, predominantly female, single and had completed a bachelor's degree. The most represented age groups were 16–25 years old. Statistically significant gender differences were found in the perception of “recognition and/or rewards received” and “making professional contacts,” among others. The primary motivations for volunteering included pleasure, enhancing personal and professional profiles, passion for the Olympic Games, and love for sport. Furthermore, it is noteworthy that 92% of volunteers expressed a willingness to participate in similar future events, which serves to underscore the extremely positive experience.

Overall, the literature reveals that the characteristics, motives and satisfaction of volunteers are shaped by a multitude of factors. Consequently, it is important to gain insight into these dynamics to develop effective volunteer management strategies and to ensure the success of MSEs.

### 2.2 Sport event volunteering in the context of special olympics

The involvement of volunteers in the organization of sport events for people with disabilities is crucial (6). In recent years, studies have been conducted on various aspects in the context of the Special Olympics (SO), the largest global sports movement for people with intellectual and multiple disabilities (27).

Long and Goldenberg (23) employed an interview-based approach to examine the characteristics and values of volunteers engaged in the San Luis Obispo County SO. The findings indicate that many volunteers were friends or family of athletes, reflecting the deeper, personal connection to the cause that characterizes volunteers in the context of the SO. Their values directly benefited the athletes, with volunteers appreciating the positive impact of sports and finding it personally rewarding to support these opportunities. Individuals who had volunteered for a period exceeding one year were predominantly parents of athletes. They placed a high value on the program for fostering competition, social interaction and independence. They associated it with health benefits, increased self-esteem, and personal fulfilment for both athletes and themselves, fostering a sense of belonging and positive relationships. Those who had volunteered for a year or less, typically without children participating in the program, were primarily driven by an interest in sports and athletes. They placed significant emphasis on the value of learning through interactions, associating it with personal growth, enhanced self-esteem, and a sense of fulfilment. Building on the research focus of motivations, Khoo and Engelhorn (28) conducted an investigation of volunteer motivations at the inaugural National SO in Ames, Iowa, utilizing the SEVMS. The results demonstrated that the primary motivating factors for volunteers were purposive incentives, including a desire to ensure the success of the event and contribute positively to the community. In addition, a factor analysis yielded a five-factor model, with the altruistic factor (purposive) being the most important. Moreover, Li and Wang (29) investigated the impact of volunteering at the 5th SO of the People's Republic of China on participants' perceptions of inclusion for individuals with intellectual disabilities. The researchers employed a measurement replication design and found that a one-week volunteering engagement significantly improved volunteers' attitudes towards inclusion. Nevertheless, no significant gender-based differences were identified. Furthermore, Hallmann et al. (30) investigated the relationship between motivations, commitment, and social capital among volunteers at the 2014 National Summer SO in Germany. The findings revealed that personal growth was the primary motivator. In addition, the study demonstrated that motivations were significantly associated with both commitment and social capital, with commitment acting as a mediator.

These studies provide valuable insights into the specific motivations and effects of volunteering for the SO, emphasizing that the decision to volunteer is influenced by a complex interplay of various factors. It is therefore evident that further research is required into this multidimensional phenomenon, particularly in the context of the SO (31).

### 2.3 Sport event volunteering and volunteering legacy

“Creating sustainable legacies is a fundamental commitment of the Olympic Movement. Every city that hosts the Olympic Games becomes a temporary steward of the Olympic Movement. It is a great responsibility. It is also a great opportunity. Host cities capture worldwide attention. Each has a once-in-a-lifetime chance to showcase the celebration of the human spirit. And each creates a unique set of environmental, social and economic legacies that can change a community, a region, and a nation forever.” (32)

This commitment by Jaques Rogge, former President of the International Olympic Committee (IOC), to long-term impact of the Olympic Movement emphasizes the importance of considering not only the immediate benefits of hosting MSEs but also their enduring effects on the host community. In terms of social legacy, the IOC also identifies the potential for fostering a culture of volunteering as a crucial aspect of social legacy (32). In consequence, an understanding of the social impact of sport events, particularly the development of a volunteering legacy, has become a significant area of research, as will be demonstrated in the following section. In this study, the term *volunteering legacy* is defined based on Preuss' definition of legacy (33): “[A]ll planned and unplanned, positive and negative, material and immaterial structures that are created in the context of volunteering for and during a sport event and that persist beyond the event.”

One illustrative example of a study focusing on the legacy of volunteering is that conducted by Doherty (34) who surveyed volunteers from the 2001 Canada Summer Games, a high-level national competition for up-and-coming Canadian athletes. The findings indicated that prospective volunteers were significantly affected by the financial costs associated with the event, including feelings of being overburdened with tasks and inconvenienced. Notwithstanding these challenges, factors such as community contribution and a positive life experience were also found to predict future involvement. In contrast, for those who provided their services on-site, future involvement was more strongly influenced by the benefits of the event, including social enrichment and community contribution. Nevertheless, personal inconvenience and an insufficient workload were also identified as significant predictors of future involvement. To further develop the concept of a volunteering legacy, Koutrou and Pappous (35) examined the impact of the Olympic Games on future volunteering. The study was conducted to gain insights into the backgrounds of volunteers to identify factors that may contribute to an improved volunteering culture following the London 2012 Games. The results demonstrated the multidimensional nature of volunteers' motivations, with “love of sport and the Olympics” and “interpersonal contact” identified as the primary motivators of the surveyed volunteers. In addition, individuals driven by a desire for interpersonal contact exhibited heightened awareness of volunteering opportunities and showed greater willingness to continue volunteering in diverse contexts, while being less inclined to give up. In a recent contribution to the field, a study utilizing an online questionnaire, conducted by Dickson et al. (36), evidenced that individuals with no prior volunteering experience were more likely to indicate that they would be willing to volunteer more after the event than those with previous experience. The study, which employed an online survey, was conducted in a post-event context, following the 2016 Rio Olympic and Paralympic Games. This finding contrasts with that of a previous study conducted by Dickson et al. (18), which examined volunteer retention at the Vancouver 2010 Olympic and Paralympic Winter Games using the same survey instrument as in 2024. In that study, individuals with prior experience of volunteering for events, sports, or community groups were less likely to indicate that they intended to volunteer again following the event. It is not uncommon for discrepancies of this nature to arise in research on volunteering and event legacies. As Dickson et al. (18) observe, existing studies often resemble a collection of case studies rather than a cohesive body of work. This is due to variations in research methods, sampling approaches, and event-specific contexts. It is therefore important to utilize similar methods and instruments to gain a deeper understanding of volunteering and volunteering legacies (18).

Despite the growing interest in volunteering at sport events and the lasting impact volunteers have, there remains a significant gap in understanding the specific factors that drive both the recruitment and retention of volunteers, particularly for events like the SO. Furthermore, existing research often fails to account for the diversity of volunteer groups involved in these events, overlooking their unique motivations and challenges. This study seeks to address these gaps by offering a more nuanced understanding of the dynamics of volunteer engagement.

## 3 Methodology

### 3.1 Research context, sample, and procedures

The study was conducted in the context of SOWG 23, the world's largest inclusive sport event, which took place in Berlin, Germany. The event encompassed 6,500 athletes competing in 26 sports and two demonstration sports, and was supported by 18,000 volunteers from 126 countries (37). This event was strategically chosen for this research because it has a unique context, which offers the potential for a substantial number of actively engaged volunteers, thereby providing valuable insights into volunteer motivations and long-term commitment. In addition, the SOWG 23 were selected on the basis of their status as a leading MSE for athletes with mental and multiple disabilities.

The data were collected via a structured online questionnaire. This approach differs from the more commonly employed method of standardized interviews (38), offering a number of significant advantages.

On behalf of the research team, the SOWG 23 Organizing Committee (OC) distributed the questionnaire by email to 10,000 accessible email addresses of volunteers who had participated in the SOWG 23. The group consisted exclusively of individuals who had been officially registered and trained as volunteers by the OC. Those with team-specific roles, such as coaches or chaperones, were categorized as part of the delegations and were not considered official volunteers of the OC. Consequently, they were not provided with the link to the questionnaire and only OC registered volunteers were surveyed. The online survey was conducted five months after the event, between November 13 and December 18, 2023. This point in time was chosen because, firstly, in the immediate aftermath of the event it is not possible to determine whether volunteers will maintain their commitment to volunteering over time; allowing a substantial period to elapse before administering the survey allows a more accurate assessment of the long-term impact of their volunteering at SOWG 23. Secondly, the possibility of conducting the survey at an even later date was constrained by data protection regulations, which limited the period during which the OC could access volunteers' email addresses. In consideration of these factors, a five-month period was deemed the most practical approach for the collection of reliable and meaningful data, thus enabling the addressing of the research question posed by this study.

### 3.2 Survey instrument

The online questionnaire was distributed via Unipark. It began with an introductory statement and a question regarding the respondent's involvement as a volunteer at the SOWG 2023. In the event of a negative response, the respondent was directed to the final page of the questionnaire. This ensured that only those who had engaged in volunteer activities at the SOWG 2023 were included.

A seven-point Likert scale was utilized to assess the level of satisfaction with the volunteer activity at the SOWG 2023, with the gradations ranging from “not at all” (1) to “completely” (7). Subsequently, expectations in relation to the volunteer activity prior to the event were recorded with 1 indicating “very low” and 7 indicating “very high.”

The motivational factors underlying engagement at the SOWG 2023 were examined using the Volunteer Motivation Scale for International Sporting Events (VMS-ISE) (39, 40). The selection of the VMS-ISE for this study is supported by its specific design to assess motivations in the context of sport event volunteering. In contrast to other scales, such as the SEVMS, the VMS-ISE incorporates distinctive factors, including “extrinsic rewards” and “love of sport,” (25) which are pivotal to the volunteer experience at sport events. Furthermore, the VMS-ISE has become the most prevalent instrument for data collection in this context in recent years, according to Angosto et al. (25), making it a robust and pertinent selection for this study. The scale was initially developed by Bang and Chelladurai (41) in the context of the 2002 FIFA World Cup. The present study employed the revised version of the scale, which was further developed and validated in other studies (39, 40). The results of the study conducted by Bang et al. (39) in the context of the Athens 2004 Olympic Games demonstrated that a seven-factor model, comprising the factors “expression of values,” “patriotism,” “interpersonal contacts,” “personal growth,” “career orientation,” “extrinsic rewards,” and “love of sport,” showed satisfactory validity and reliability. This serves to confirm the applicability of the questionnaire for measuring the motivation of volunteers in international sport events. Moreover, the scale employed in this study was expanded to include the factor “tradition,” which can be found in the study by Dickson et al. (19), as well as the factors “entertainment” and “recognition” as a result of theoretical considerations. As part of the study, the structure of the revised VMS-ISE was subjected to an exploratory factor analysis in order to ascertain its validity. Both the Bartlett's test [*χ*^2^(820) = 11068.58, *p* < .001] and the Kaiser-Meyer-Olkin measure of sampling adequacy (KMO = .891) indicate that the variables are suitable for factor analysis. A principal component analysis with varimax rotation was conducted, resulting in the identification of nine factors with eigenvalues exceeding 1.0. However, given the proximity of an additional factor with an eigenvalue of 0.95 to 1.0 and the influence of theoretical considerations, a ten-factor solution was selected, accounting for 67.5% of the variance. The items measuring the motives were presented in a randomized order, and respondents were required to rate each item on a seven-point Likert scale, where 1 represented “not applicable at all” and 7 represented “completely applicable.”

To ascertain the level of participation in voluntary activities before and after the SOWG 23, respondents were initially asked to specify whether they had engaged in any volunteer activities within pre-defined 24 specified domains (sports clubs, small sports events, educational institutions, etc.) at any point prior to the event. If they responded in the affirmative to one or more of the domains, they were then asked to provide further detailed information regarding their pre-event involvement. This included, for instance, the length of the commitment and the number of working hours per week. The objective of recording the history of volunteering after the event was to determine the temporal period between the conclusion of the event and the administration of the survey. The questions were based on those related to volunteering prior to the event. Additionally, respondents were asked whether their current volunteering activity could be attributed to their experience of volunteering at the SOWG 23.

The intention to continue volunteering after the SOWG 23 was evaluated in relation to specific areas, including involvement in sports clubs or future sport events. Additionally, statements were included to assess awareness of volunteering and general interest in volunteer activities. Overall, the intention to volunteer in the future was evaluated using nine statements, each rated on a seven-point Likert scale, where 1 indicated a “strong decrease” and 7 indicated a “strong increase.”

To ascertain which aspects of the event itself were perceived as particularly attractive in terms of sport-event-volunteering activities at the SOWG 2023, the survey instrument included eleven specific features of the sport event, such as the uniqueness of the event or being part of the volunteer community. A seven-point Likert scale was employed for this purpose, with 1 representing “not attractive at all” and 7 representing “very attractive,” to ascertain which sport event characteristics are perceived as particularly attractive and may foster long-term engagement.

The socio-demographic variables collected included parental volunteer history, gender, age, marital status, presence or absence of children, and highest level of education. These variables were selected based on their potential influence on the volunteers' motivational structure, satisfaction, and intention to volunteer again, as evidenced by previous studies (18, 36). In consideration of the extant literature, the variable of the presence or absence of children was included, given the emphasis placed on the importance of family involvement in the context of volunteering at the SO (23).

### 3.3 Data analysis

Once the data collection phase had been completed, the data were transferred to IBM SPSS Statistics 27 for descriptive and inferential statistical analyses. Furthermore, the responses on the seven-point Likert scale were aggregated for the purposes of descriptive analysis. To facilitate interpretation and highlight meaningful trends, the responses on the seven-point Likert scale were aggregated in the descriptive part of the analysis. The aggregation was conducted for categories 1 to 3 and for categories 5 to 7. Category 4 was treated as the neutral category. A total of 515 completed questionnaires were received. However, three participants who indicated that they were not active at the SOWG 23 were excluded, resulting in a final sample size of *n* = 512 for the comprehensive analyses. The aim of this was to elucidate the motives and characteristics of SOWG 23 volunteers. Subsequently, a group-specific analysis was conducted, whereby the participants were classified into three groups based on their individual volunteering history. Group 0 (“active before and after”), consisted of 399 volunteers who were active both prior to and following the event. Group 1 (“active”) comprised 83 volunteers who had not been active directly prior to the event but subsequently became active because of the event. It is important to note that these individuals may have previously engaged in volunteer activities at some point in their lives. Group 2, designated “not active,” comprised 30 volunteers who had not been active prior to the event and did not become active because of the event. The group-specific analysis was conducted on the two groups “active” (group 1) and “not active” (group 2). The “active before and after” (group 0) was deemed less pertinent to the research question, as these volunteers demonstrated a consistent pattern of volunteering both before and after the event. This made it challenging to ascertain how the event specifically influenced their volunteering activities. As a result, the “active before and after” group was excluded from the group-specific analysis. By focusing on the “active” and “not active” groups, a more precise distinction was made between volunteers whose engagement was directly influenced by the SOWG 23 and those whose involvement remained negative and thus unaffected by the event. This allowed for a more concentrated investigation of the factors influencing engagement and the potential for sustained dedication extending beyond the event.

## 4 Results

The initial section of this chapter presents the results for the overall sample of surveyed volunteers, providing an overview of the characteristics and key motivations of SOWG 23 volunteers. This is followed by a detailed analysis of specific groups. The group-specific analysis allows for the identification of distinct differences and similarities within the volunteers of the SOWG 23, offering deeper insights into how various factors may influence long-term volunteer commitment and thus creating a volunteering legacy.

### 4.1 Overall sample

#### 4.1.1 Sociodemographic variables

Table 1 provides an overview of the predominant sociodemographic characteristics of the overall sample. The data indicate that the majority of the surveyed SOWG 23 volunteers were female (62.1%), married or in a life partnership (56.1%), without children (51.2%), held a university degree or higher qualification (56.1%), and were, on average, 47.9 years old. Most respondents (53.5%) indicated that their parents were not engaged in voluntary work. Furthermore, most respondents (91%) had prior experience of volunteering outside of sport events prior to participating in SOWG 23. This experience was particularly prevalent in sports clubs (53.7%), educational settings (44.9%), and working with people with disabilities (40.8%).

**Table 1:** Sociodemographic variables of total sample, group “active” and group “not active” (source: own presentation).

Sociodemographic characteristics		Total sample (*n* = 512)	Group “active” (*n* = 83)	Group “not active” (*n* = 30)
Gender	Male	35.7%	42.2%	40.0%
	Female	62.1%	55.4%	56.7%
	Diverse	0.4%	0.0%	0.0%
	Not specified	1.8%	2.4%	3.3%
Marital status	Single	35.0%	34.9%	30.0%
	Married/Life partnership	56.0%	56.6%	43.3%
	Not specified	9.0%	8.5%	26.7%
Have children	No	51.2%	48.2%	66.7%
Educational qualification	No school leaving certificate (yet)	2.3%	0.0%	13.3%
	Secondary school leaving certificate	2.0%	3.6%	3.3%
	Comprehensive school leaving certificate	12.6%	16.9%	20.0%
	High school diploma	27.0%	27.7%	16.7%
	University degree or higher	56.1%	51.8%	46.7%
Parental volunteer history	Parents do no voluntary work	53.5%	62.7%	83.3%
Previous volunteering experience (beyond sport events)	Ever volunteered	91.0%	87.9%	0.0%
Mean Age		47.9	49.8	43.6

#### 4.1.2 Motives

To identify the primary motives influencing volunteers' engagement with SOWG 23, ten key motives were subjected to analysis (“expression of values,” “patriotism,” “interpersonal contacts,” “career orientation,” “personal growth,” “extrinsic rewards,” “love of sport,” “tradition,” “entertainment,” “recognition”). The motive “expression of values” was rated as applicable by 91.9% of respondents, representing the highest proportion among all motives (see Table 2). Only 3.1% of respondents indicated that this motive did not apply. The motive “interpersonal contacts” was also relevant, with 70.7% of respondents rating it as applicable and 14.5% rating it as not applicable. In contrast, “extrinsic rewards” was deemed applicable by 6.0% of participants, with 88.1% indicating that it did not apply. This represents the highest percentage for this rating level. The subsequent motive was that of “recognition,” which was deemed applicable by 14.7% of respondents and was not applicable by 73.3% of respondents. A detailed distribution of responses for all motives can be found in Table 2.

**Table 2:** Response distribution of motives measured on a Likert scale from 1 (“not applicable at all”) to 7 (“completely applicable”) of the total sample, group “active” and group “not active” (source: own presentation).

	Total Sample			Group “active”			Group “not active”		
Motive	Not applicable (1–3)	Neutral (4)	Applicable (5–7)	Not a. (1–3)	Neut. (4)	Appl. (5–7)	Not a. (1–3)	Neut. (4)	Appl. (5–7)
Expression of values	3.1%	5.0%	91.9%	2.4%	4.1%	93.5%	7.5%	5.2%	87.3%
Patriotism	49.0%	17.5%	33.4%	38.8%	18.1%	43.1%	48.7%	17.3%	34.0%
Interpersonal contacts	14.5%	14.8%	70.7%	11.2%	9.6%	79.2%	12.5%	12.4%	74.1%
Career orientation	59.7%	9.7%	30.6%	51.3%	12.1%	36.6%	56.7%	10.0%	33.3%
Personal growth	27.1%	14.6%	58.3%	18.7%	13.0%	68.3%	28.3%	17.5%	54.2%
Extrinsic rewards	88.1%	5.9%	6.0%	85.8%	7.7%	6.5%	88.0%	7.3%	4.7%
Love of sport	25.2%	13.2%	61.6%	19.3%	11.4%	69.3%	35.0%	20.0%	45.0%
Tradition	65.9%	10.3%	23.8%	62.7%	10.2%	27.1%	79.1%	5.9%	15.0%
Entertainment	29.5%	11.3%	59.2%	24.8%	12.1%	63.1%	28.9%	10.0%	61.1%
Recognition	73.3%	12.0%	14.7%	64.5%	18.6%	16.9%	81.7%	5.0%	13.3%

### 4.2 Group specific analysis

#### 4.2.1 Sociodemographic variables

The group-specific analyses pertaining to socio-demographic variables (see Table 1) indicate that the majority of respondents in both groups were female, without children, married or in a civil partnership, had attained a university degree or higher, and had parents with no history of volunteering. The mean age was 49.8 years for the “active” group and 43.6 years for the “not active” group. Moreover, most members of the “active” group had prior experience of volunteering outside of the context of sports events prior to SOWG 23.

Regarding post-event volunteering, 47% of respondents in the “active” group were engaged in other MSEs, 39.8% were also or solely involved in smaller sport events such as marathons, and 37.3% participated in work with people with disabilities.

A Chi-square test was conducted to ascertain whether there were significant differences between the two groups in relation to their socio-demographic variables. The analysis revealed statistically significant associations between group membership and “parental volunteering history” [*χ*^2^(1) = 4.342, *p* = .037, V = .196], “marital status” [*χ*^2^(2) = 6.412, *p* = .041, V = .24], and “educational qualifications” [*χ*^2^(4) = 12.394, *p* = .015, V = .331]. To sum up the results indicate significant differences between the “active” and “not active” groups in terms of “parental volunteering history,” “marital status,” and “educational qualifications.”

#### 4.2.2 Satisfaction and expectations

The group-specific analysis of satisfaction and expectations among SOWG 23 volunteers shows that 95% of the “active” group and 83.3% of the “not active” group reported high satisfaction with their volunteering experience, while 2.4% and 6.7%, respectively, indicated low satisfaction. A neutral response was exhibited by 2.4% of the “active” group and 20.5% of the “not active” group. Concerning expectations, 72.3% of the “active” group and 50% of the “not active” stated that they had high expectations, whereas low expectations were expressed by 7.2% of the “active” group and 13.3% of the “not active” group. Neutral expectations were noted by 20.5% of the “active” group and 36.7% of the “not active” group.

A Mann–Whitney *U*-test revealed no statistically significant difference in satisfaction between the “active” (median = 7.0, *n* = 83) and “not active” (median = 6.0, *n* = 30) groups, *U* = 1,064, *Z* = −1.286, *p* = .199, *r* = .12. However, a significant difference in expectations was found between the “active” (median = 5.0, *n* = 83) and “not active” (median = 4.5, *n* = 30) groups, *U* = 948.500, *Z* = −1.976, *p* = .048, *r* = .19. It can be concluded that while satisfaction levels did not significantly differ between the groups, expectations were significantly higher in the “active” group.

#### 4.2.3 Motives

The results of the motives for engagement of the surveyed volunteers at the SOWG 23 demonstrate that the motive “expression of values” was rated as applicable by 93.5% of the “active” group and 87.3% of the “not active” group, representing the highest percentage at this rating level. A mere 2.4% of the “active” group and 7.5% of the “not active” group indicated that it was not applicable. In contrast, “extrinsic rewards” was deemed applicable by 6.5% of the “active” group and 4.7% of the “not active” group, while 85.8% of the “active” group and 88% of the “not active” group responded that it was not applicable. This was the highest percentage at this rating level among both groups for all analyzed motives. A detailed overview of the group-specific response distribution for all analyzed motives is provided in Table 2.

Chi-square tests were conducted to ascertain whether there were significant differences between the two groups in relation to their motivation. The analysis revealed a significant difference between the “active” and “not active” groups regarding the motive “love of sport” [*χ*²(7) = 16.04, *p* = .025, *V* = .67]. In particular, 69.3% of the “active” group indicated that the “love of sport” motive was applicable for their engagement, whereas 54% of the “not active” group responded in the same manner. It can be posited that the “love of sport” factor plays a significantly more prominent role in motivating the “active” group.

#### 4.2.4 Post-event volunteering intention

With regard to post-event intentions to volunteer, general interest in volunteering, and awareness of volunteering opportunities, a decrease was reported in the “intention to volunteer in sports associations” by 10.8% of respondents in the “active” group and 13.3% of volunteers in the “not active” group. This represents the highest proportion for this rating level within both groups. In contrast, 95.2% of volunteers in the “active” group indicated an increased “intention to volunteer at a mega sport event again”, representing the highest percentage for this item within group “active.” In the group “not active,” the item “awareness of the range of volunteering opportunities” was rated as an increase by 86.7% of respondents, representing the largest proportion at this rating level within this group. A comprehensive illustration of the responses is presented in Table 3.

**Table 3:** Response distribution post-event intentions to volunteer, general interest in volunteering, and awareness of volunteering opportunities measured on a Likert scale from 1 (“strong decrease”) to 7 (“strong increase”) of group “active” and group “not active” (source: own presentation).

After volunteering at a sports event, my…	Group “active”			Group “not active”		
	Decrease (1–3)	Neutral (4)	Increase (5–7)	Decrease (1–3)	Neutral (4)	Increase (5–7)
General Intention of volunteering	1.2%	19.3%	79.5%	3.3%	26.7%	70.0%
Intention to volunteer at a mega sport event again	2.4%	2.4%	95.2%	3.3%	16.7%	80.0%
Intention to volunteer at a minor sport event	2.4%	24.2%	73.4%	3.3%	36.7%	60.0%
Intention to volunteer at a club sport event	8.4%	37.3%	54.3%	6.6%	43.4%	50.0%
Intention to volunteer in sports clubs	8.4%	42.3%	49.3%	10.0%	60.0%	30.0%
Intention to volunteer in sports associations	10.8%	47.0%	42.2%	13.3%	43.3%	43.4%
Intention to volunteer in the non-sports sector	3.6%	39.8%	56.6%	3.3%	26.7%	70.0%
General interest in Volunteering/Community Service	0.0%	24.1%	75.9%	3.3%	16.7%	80.0%
Awareness of the range of volunteering opportunities	1.2%	15.7%	83.1%	3.3%	10.0%	86.7%

A Mann–Whitney *U*-test was employed to ascertain whether there were significant differences in intention, general interest in volunteering, and awareness of volunteering opportunities between the two groups. The analysis revealed a statistically significant difference in the “intention to volunteer at a mega sport event again” (*U* = 790.5, *Z* = −3.25, *p* = .001, *r* = .31) between the “active” group (median = 7.0, *n* = 83) and the “not active” group (median = 6.0, *n* = 30). It can be concluded that there is a significantly higher “intention to volunteer at a mega sport event again” among volunteers in the “active” group compared to those in the “not active” group.

#### 4.2.5 Sport event characteristics

The response distribution, regarding the appeal of the characteristics of sport events, as illustrated in Table 4, shows that 22.9% of the volunteers in the “active” group and 46.7% in the “not active” group indicated that the aspect of “receiving recognized certificates” was not attractive, representing the highest percentage at this rating level in both groups. Only 22.9% in group “active” and 33.3% in group “not active” rated it as attractive. 84.3% of the “active” group rated the “uniqueness of the event” as attractive, indicating the highest percentage for this rating level within this group. A mere 9.6% of this cohort found it unattractive. In contrast, the highest rating level for the attractiveness of a characteristic in the group “not active” was found to be 96.7% for “being part of the volunteer community,” with 3.3% indicating that they found this characteristic not attractive.

**Table 4:** Response distribution of sport event characteristics measured on a Likert scale from 1 (“very attractive”) to 7 (“not attractive at all”) of group “active” and group “not active” (source: own presentation).

	Group “active”			Group “not active”		
Characteristic	Not attractive (1–3)	Neutral (4)	Attractive (5–7)	Not attractive (1–3)	Neutral (4)	Attractive (5–7)
The uniqueness of the event	9.6%	6.1%	84.3%	3.3%	6.7%	90.0%
The fixed period of the assignment	8.4%	10.9%	80.7%	6.7%	6.7%	86.6%
The short duration of the assignment (1–4 weeks)	16.8%	12.0%	71.1%	10.0%	23.3%	66.6%
The clearly defined area of responsibility	18.0%	18.1%	63.9%	10.0%	10.0%	80.0%
The volunteer selection process	48.1%	24.3%	27.6%	33.3%	20.0%	46.7%
Targeted preparation for my volunteer work	9.6%	22.9%	67.5%	10.0%	13.3%	76.7%
Receiving recognized certificates	56.6%	20.5%	22.9%	46.7%	20.0%	33.3%
Applying via an online platform	24.0%	15.7%	60.3%	16.7%	16.7%	66.6%
Being part of the volunteer community	10.8%	9.6%	79.6%	3.3%	0.0%	96.7%
That I am assigned a direct contact person	27.6%	16.9%	55.5%	16.7%	13.3%	70.0%
That I am appreciated	19.2%	16.9%	63.9%	26.7%	23.3%	50.0%

A Mann–Whitney *U*-test was conducted to ascertain whether there were significant differences between the groups in their evaluations of the appeal of the characteristics associated with sport events. The results demonstrated no statistically significant differences. It can thus be concluded that there are no significant discrepancies between the “active” and “not active” groups in their perceptions of the attractiveness of the analyzed characteristics in the context of SOWG 23.

#### 4.2.6 Regression analysis of factors predicting group membership

To identify the influence of “parental volunteering history,” “marital status,” “educational level,” “expectations,” “intention to volunteer at mega sport events again,” and “love of sport” on the probability of group membership in the “active” and “non-active” groups a binomial logistic regression was conducted. These variables were deliberately selected based on their identification as significant in the preceding analyses, which contributed to the prediction of the likelihood of (re)volunteering after the event and in turn, to the creation of a sustainable volunteering legacy.

Due to multicollinearity, as indicated by the variance inflation factor value, the motive “love of sport” was excluded from the regression model. The resulting regression model, including the variables “parental volunteering history,” “marital status,” “educational level,” “expectations,” and “intention to volunteer at a mega sport event again,” was statistically significant [*χ*^2^(18) = 48.01, *p* < .001, *n* = 113], resulting in a large amount of explained variance as shown by Nagelkerke's *R*^2^ = .51. The model's classification accuracy was 83.19%, with a sensitivity of 93.98% and a specificity of 53.3%. Of the variables included, “intention to volunteer at a mega sport event again” contributed significantly to the prediction of group membership for Likert scale scores of 4 (*B* = −3.98, OR = .02, *p* = .002) and 6 (*B* = −2.03, OR = .13, *p* = .006). Furthermore, the “parental volunteering history” was identified as a significant predictor of group membership.

In summary, the results of the binomial logistic regression indicate that the probability of belonging to the “active” group is significantly lower for respondents who rated their “intention to volunteer at a mega sport event again” as 4 or 6 on the Likert scale. Moreover, respondents with parents who had no history of volunteering exhibited a significantly lower probability of belonging to the “active” group (*B* = −1.61, *R* = .2, *p* = .039).

## 5 Discussion

The aim of this study was to identify the factors that facilitate long-term volunteering and promote sustainable engagement, particularly in the context of SO. To answer the research question, the key findings will now be discussed, followed by practical implications, limitations and suggestions for future research. For a more detailed discussion, the following chapter divides the research question into three parts:

1. Who are the SOWG 23 volunteers?
2. What motivations drive volunteers to participate in the SOWG 23?
3. What factors contribute to the long-term commitment of volunteers for the SOWG 23?

### 5.1 Who are the SOWG 23 volunteers?

The findings revealed that the 512 volunteers surveyed were predominantly female, older, and experienced in volunteering. This pattern is consistent with the findings of previous studies in this field (18, 19). Moreover, the relatively high number of female respondents provides a potential indication that women may evince a greater motivation in engaging in volunteer activities at sport events than men (21, 30). The high proportion of female respondents, particularly in the context of the SOWG 23, may be attributed to the notion that women are more strongly motivated by value-oriented factors, whereas men are more likely to prioritize instrumental attitudes and goals (42). In the case of the SOWG 23, which is characterized by a strong focus on inclusion, community and social values (27), these value-driven motivations could play a decisive role in determining whether or not an individual engages with the event. Although these findings align with previous studies, they are specific to the respondents and context of this research, and thus caution should be exercised when attempting to generalize.

Moreover, most of the respondents are married or in a civil partnership (56.1%). This may be indicative of the particular family-related dynamics associated with the SO (23). The act of volunteering can be perceived by participants as an opportunity to promote the values and goals of the event, particularly those related to support, care, and inclusion (27). These values often align with those held by families. The event thus provides a framework in which family and altruistic values can be expanded in a structured way.

The socio-demographic profile of the SOWG 23 volunteers surveyed displays a notable degree of consistency with that of volunteers at other MSEs. These findings offer valuable insights that can inform the design of future volunteering initiatives and recruitment strategies.

### 5.2 What motivations drive volunteers to participate in the SOWG 23?

The results of the surveyed volunteers at the SOWG 23 provide compelling evidence that intrinsic factors are the primary drivers of volunteer engagement at this event, particularly the “expression of values” (see Table 2). This finding is consistent with existing literature, which frequently demonstrates that the “expression of values” associated with altruism is a dominant motivator among volunteers (25, 40). The study identified the high importance of the “expression of values” among the volunteers, which reflects their motivation to become involved in a meaningful cause. In the context of the SOWG, which stand for the promotion of inclusion and self-determination (27), among other things, this relevance is particularly emphasized. Similarly, the category of “interpersonal contacts” emerged as a noteworthy motivator, underscoring the influence of social interactions in shaping favorable volunteer experiences. This finding corroborates prior research indicating that volunteering at international events offers distinctive opportunities to connect with individuals who share similar interests and develop meaningful relationships (40). The dynamic and inclusive atmosphere at SOWG 23 likely served to accentuate the importance of these social connections, thereby fostering a sense of belonging and shared purpose among volunteers. This highlights the importance of considering the social dimension of volunteering in recruitment strategies, as it has the potential to enhance the immediate experience and promote long-term engagement. In contrast, extrinsic incentives, such as material rewards, appear to have minimal motivational value for SOWG 23 volunteers. This aligns with existing research on volunteer motivations (25). Although tangible benefits such as complimentary food or t-shirts, do not represent the primary motivators for engagement, they can nevertheless exert a positive influence on volunteer satisfaction by acknowledging their contributions to the mission and values upheld by the SO, as observed by Preuss and Kebernik (43).

The collective findings highlight the pivotal role of intrinsic motivations, particularly the alignment between individual values and the mission of the event, in fostering long-term volunteer engagement.

### 5.3 What factors contribute to the long-term commitment of volunteers for the SOWG 23?

The group-specific analysis of socio-demographic variables revealed significant correlations between group membership and a number of factors, including “parental volunteering history,” “marital status,” and “educational qualification.” These findings illustrate the considerable impact of socio-demographic factors on volunteering behavior, demonstrating the multifaceted influences that shape volunteer engagement. These findings are consistent with those of previous studies, which have highlighted the importance of personal backgrounds in influencing the trajectory of volunteering (44). These insights are especially valuable, as they indicate that an individual's personal background can play a pivotal role in determining long-term volunteering patterns and can therefore inform recruitment strategies.

Regarding satisfaction and expectations, both groups reported high levels of satisfaction, with the “active” group (median = 7.0) exhibiting slightly higher levels than the “not active” group (median = 6.0), though without statistical significance (*p* = .199). Nevertheless, a weak statistically significant difference was observed in expectations (*p* = .048), with the “active” group (median = 5.0) reporting higher expectations than the “not active” group (median = 4.5). The discrepancy may be attributed to the fact that the volunteers in the “active” group had previously engaged in volunteering activities, which may have shaped their expectations. Nevertheless, the considerable overall satisfaction levels indicate that, despite the existence of disparate expectations, the event was ultimately able to meet the expectations and motivations of most respondents. However, it is important that the role descriptions are transparent and that expectations are managed effectively, as this can encourage long-term loyalty (45).

The analysis of motivational factors revealed a significant difference between the two groups regarding the motive “love of sport” (*p* = .025, *V* = .67), with this motive playing a more important role in the “active” group. This result offers one potential explanation for the post-event commitment of volunteers in the “active” group. Their passion for sports appears to be a driving force behind their sustained engagement, serving as a foundational motivation that encourages them to remain involved in volunteer activities after the event. Therefore, enthusiasm for the sport can be seen to foster not only initial participation but also a longer-term commitment to volunteering in related contexts.

In terms of post-event intentions to volunteer, general interest in volunteering, and awareness of volunteering opportunities, the findings indicate a markedly higher incidence of “intention to volunteer at a mega sport event again” (*p* = .001) among the “active” group (median = 7.0) in comparison to the “not active” group (median = 6.0). This observation is indicative of the event's capacity to foster sustained volunteer engagement. This is further corroborated by the fact that 46.9% of this group have already engaged in volunteer activities at another MSE subsequent to their involvement at the SOWG 23. This is consistent with earlier findings on the positive effects of such events on future engagement (21). It is additionally noteworthy that 37.3% of the group “active” proceeded to engage in voluntary activities for people with disabilities. The evident enthusiasm for working with people with disabilities following the event underscores the necessity of considering the distinctive attributes of each event when recruiting volunteers for the sports and non-sports sector. In the context of our study, it can be postulated that volunteers may be more inclined to engage in work with individuals with disabilities than in areas such as education, as evidenced by their participation in the SOWG 23, which has demonstrated their interest and enthusiasm to volunteer in this field.

With regard to the appeal of event characteristics, both groups identified the event's distinctive features, its fixed period, and the sense of community as attractive aspects. Nevertheless, the preference for the “uniqueness of the event” among the “active” group and “being part of the volunteer community” among the “not active” group demonstrates a divergence in perception between the two groups (see Table 4). Despite these group-specific differences, no statistically significant differences were identified. However, this discrepancy underscores the necessity of implementing tailored volunteer recruitment strategies designed to cater to the specific interests of each group, with the objective of enhancing long-term engagement.

With respect to the results of the logistic regression, it is particularly noteworthy that the probability of belonging to the “active” group is found to decrease significantly when respondents indicate that their parents were not involved in voluntary work. Thus, the probability of belonging to the “active” group significantly increases when the respondents report that their parents had a history of volunteering. This highlights the importance of parental role models in fostering voluntary engagement. A substantial body of evidence from previous research indicates that parental volunteering has a positive impact on children, particularly during adolescence [e.g., (46)]. However, the results of the present study demonstrate that this influence may persist into adulthood. With an average age of 49.8 years in the “active” group, the findings suggest that parental volunteering may continue to shape long-term volunteer engagement, potentially through values and behaviors instilled during childhood. This enduring influence may have been further reinforced by the positive experiences of volunteering at the SOWG 23, which could have supported participants' decision to (re)engage in volunteering. To summarize, the results of the group-specific analysis of the factors that influence the long-term commitment of SOWG 23 volunteers as a result of the event indicate that a variety of factors can have a decisive influence on the decision-making process. This underscores the need for diverse volunteer recruitment strategies to attract a broad target group and sustain the enthusiasm generated during the event across both sports and non-sports organizations.

### 5.4 Practical implications

The following section presents the study's findings and their practical implications, with the objective of enhancing the recruitment and long-term retention of volunteers in the context of MSEs.

1. Tailoring communication strategies: In light of the socio-demographic variables, it is essential that organizations implement different communication strategies to engage both older, experienced volunteers and younger individuals without prior volunteering experience. For example, Generation Z may respond well to mobile apps and social media platforms that provide automated notifications about new, short-term, or recurring projects. Direct outreach *via* smartphones can increase visibility and engagement by placing volunteering opportunities directly in front of the target audience. A review of the literature reveals that Generation Z is keen to engage in volunteering activities, yet their expectations differ from those of older age groups (47). Recognizing these varying preferences, especially regarding communication channels, is crucial to fostering long-term involvement. Additionally, offering one-off projects can attract those hesitant to commit long-term, reflecting the trend toward more flexible volunteering (9). The implementation of concise, targeted initiatives can facilitate the creation of a sense of uniqueness, a fixed duration, and a sense of community among volunteers. These aspects of MSEs have been shown to be appealing to volunteers and can facilitate the establishment of sustained volunteer involvement.
2. Setting clear expectations: The findings on satisfaction and expectations indicate that higher expectations among the “active” volunteers may be associated with prior volunteering experience. Organizations should prioritize establishing clear and realistic expectations during the onboarding process for new volunteers, particularly those with prior experience. By effectively communicating roles and responsibilities, organizations can enhance volunteer satisfaction and thus promote long-term loyalty (45). A transparent onboarding process, in conjunction with training and support programs, can further enhance volunteers' engagement and motivation to remain active.
3. Focusing on intrinsic motivators: Intrinsic motives such as “expression of values” and “interpersonal contacts” played a key role in volunteer engagement at the SOWG 23. Recruitment campaigns should emphasize intrinsic motivators by aligning volunteering opportunities with personal values and societal contributions. Additionally, creating opportunities for social interaction, like team-building and networking events, can increase the appeal of volunteering. While “recognition” was less significant compared to intrinsic motivators, acknowledging volunteers' efforts remains important, as many feel their contributions are overlooked (9). Overall, focusing on intrinsic motivators and showing appreciation for volunteers' unique contributions can maintain enthusiasm and encourage long-term commitment.
4. Aligning with event Characteristics: It is essential that recruitment strategies take the distinctive characteristics of each event into account. The study illustrates that numerous volunteers who became involved because of their participation in the SOWG 23 subsequently engaged in activities related to individuals with disabilities. This demonstrates the importance of adapting recruitment strategies to align with the specific attributes of each event and targeting the interests and motivations of potential volunteers accordingly. In this context, the answer to the following question may be crucial: What influence do the specific characteristics of the event have on the background and motivation of volunteers and, consequently, on the areas from which they are most likely to be recruited?

In conclusion, effective recruitment strategies should consider the diverse profiles of volunteers and their specific needs, while also recognizing the unique characteristics of sport events. While there are certain similarities between volunteers and events, it is ultimately the distinct motivations and preferences that shape both.

### 5.5 Limitations

While the study yielded valuable insights into characteristics, motives, and factors that promote long-term volunteering, it is important to acknowledge certain limitations.

In terms of representativeness, it is important to note that the questionnaire was distributed to 10,000 email addresses known to the SOWG 23 OC, and 515 responses were received, which corresponds to a response rate of only 5.2%. The relatively low response rate gives rise to concerns regarding the representativeness of the overall sample. This may be attributed to the temporal discrepancy between the event and the data collection, during which a considerable number of volunteers may have become disengaged from the event, resulting in a reduced response rate. However, this timing was essential for assessing the long-term evolution of initial enthusiasm and commitment. Furthermore, it is typical for online surveys to have lower response rates than other methods (48). It is also noteworthy that the majority of respondents were actively engaged in volunteering activities prior to the event and continued to do so after the event. This suggests that the survey may have attracted those with a high level of commitment. This self-selection may have biased the results by overrepresenting highly satisfied and engaged volunteers, thereby limiting the generalizability of the findings to other contexts. Furthermore, the SOWG 23 represents a distinctive form of sport event, exhibiting notable differences from other MSEs. It is possible that the findings may not be applicable to other types of sport events or volunteer activities due to their distinctiveness.

However, for the purposes of differentiating between the “active” and “not active” groups, the return rate was not a crucial factor, as the differentiation was conducted within the sample using specific factors. Therefore, the results were found to be reliable and the only critique could be the small sub-sample size of *n* = 30 in group “not active.”

To address these limitations, further research should incorporate a broader range of mega and major sport events, using the questionnaire developed for this study to test the generalizability of these results, validate the findings of the current study, and gain a more comprehensive understanding of the factors that support long-term volunteer engagement.

## 6 Conclusion

Extensive research has explored the characteristics, motivations, and outcomes of sport event volunteers, particularly in inclusive settings involving individuals with and without disabilities (18, 21, 30). However, significant gaps remain in understanding the factors that influence volunteer recruitment and long-term retention, especially in the context of the SO. This study addressed this gap by analyzing the characteristics of volunteers through a structured questionnaire, with a view to identifying the key factors that promote long-term engagement and therefore contribute to the research field of sustainability and volunteer legacy.

The findings effectively address the research question regarding the characteristics, motivations, and factors influencing long-term commitment among SOWG 23 volunteers. The socio-demographic data reveal that the surveyed volunteers are broadly similar to those from other MSEs, being predominantly female, older, and possessing prior volunteer experience. In terms of motivations, the results underscore the pivotal role of intrinsic factors in engaging volunteers at the SOWG 23, while extrinsic rewards have a comparatively lesser influence. Furthermore, the analysis demonstrates that a multitude of factors influence the decision to be active because of the event, thereby illustrating the complex interplay of influences. Moreover, validation and expansion of the VMS-ISE through the incorporation of “entertainment” and “recognition” factors provide valuable insights into the motivations of volunteers at MSEs.

From a practical standpoint, the study highlights the need for differentiated communication strategies, especially through digital channels, and the provision of flexible, project-based roles for Generation Z. Besides that, volunteers must be provided with clear task definitions, realistic expectations, and consideration of their intrinsic motivations to foster enduring satisfaction and commitment. Furthermore, when recruiting new volunteers, it is essential to consider the distinctive features of each sport event.

This study makes an important contribution to existing research by identifying specific factors that facilitate a sustainable volunteering legacy through the differentiation of volunteers within an event based on their volunteering history. It therefore plays a key role in addressing the research gap. It would be beneficial for future research to adopt a comparable methodology to substantiate these findings and further refine recommendations for sports organizations to cultivate MSE volunteers into long-term volunteers (within the sports and non-sports sector). Moreover, the administration of surveys at two distinct time points, namely immediately following the event and at a subsequent phase, could facilitate a more profound understanding of the transformation of intentions and commitments over time. This would, in turn, permit the formulation of more detailed, long-term engagement strategies.

## Data Availability

The raw data supporting the conclusions of this article will be made available by the authors, without undue reservation.
